# Metabolomics in Pathogenic Pathways and Targeted Therapies for Diabetic Neuropathy: A Comprehensive Review

**DOI:** 10.3390/metabo15020086

**Published:** 2025-02-01

**Authors:** Cornelia Bala, Adriana Rusu, Dana Mihaela Ciobanu, Gabriela Roman, Anca Elena Crăciun

**Affiliations:** Department of Diabetes and Nutrition Diseases, “Iuliu Hatieganu” University of Medicine and Pharmacy, 400006 Cluj-Napoca, Romania; cbala@umfcluj.ro (C.B.); adriana.rusu@umfcluj.ro (A.R.); groman@umfcluj.ro (G.R.); anca.craciun@umfcluj.ro (A.E.C.)

**Keywords:** metabolomics, diabetes mellitus, diabetic neuropathies

## Abstract

Introduction and objective: This literature review aims to provide an overview of the progress in metabolomic assessment in animal and cell models and in humans with diabetic neuropathy (DN). Methods: Metabolomics has emerged as an important approach for investigating, identifying, and describing biomarkers related to DN. None has yet been validated for use in clinical practice. Results: DN induced significant alterations in energy metabolism and carbohydrates, lipids, amino acids, peptides, and proteins. Several treatments for DN, evaluated using metabolomics, were proved to have promising results. Conclusions: The ideal metabolite or set of metabolites that could be used as biomarkers should identify patients with diabetes prone to develop DN or those prone to progress to severe forms of sensory loss, associated with risk of ulcerations and amputation. Another potential use of a metabolite might be as an indicator of treatment response in clinical trials using agents with potential disease-modifying properties.

## 1. Introduction

Diabetic neuropathies (DN) are the most prevalent chronic complications of diabetes occurring in 50% of patients with both type 1 and type 2 diabetes; they affect different parts of the nervous system and have diverse clinical manifestations [1,2]. DN was the fourth among the ten neurological conditions with the highest age-standardized disability-adjusted life years in 2021, being surpassed only by stroke, neonatal encephalopathy, migraine, Alzheimer’s, and other dementias [3].

The most common form of diabetic neuropathy is peripheral polyneuropathy, accounting for approximately 75% of diabetic neuropathies and resulting in increased risk of ulceration and amputation, as well as decreased quality of life in patients with neuropathic pain [1,4]. More recent data describe different sensory profiles that can display variable pain-related sensory symptoms and signs and are related with progressive severity of DN and hence progressive loss of fiber density occurring in healthy, thermal hyperalgesia, mechanical hyperalgesia, and sensory loss subgroups [5,6]. It was hypothesized that these phenotypes might indicate different classes of neurobiological mechanisms, and different responses to treatment [5]. 

Autonomic neuropathies are another group of DN among which the best-described is cardiac autonomic neuropathy (CAN) occurring in 44% and 60% of patients with long-standing type 1 and type 2 diabetes, respectively. CAN is associated with risk of arrhythmia and sudden death and is a contributor to increased heart failure risk in diabetes [7].

The pathogenesis of DN is complex and several mechanisms related to chronic hyperglycemia, and more recently dyslipidemia, are considered to contribute to DN through impairment in the structure and function of neurons, their supportive glia, the Schwann cells (SCs), and vascular and connective tissues. The role played by the nerve insulin-resistance, microvascular damage and hyperglycemia-induced polyol and hexosamine pathway activation, protein kinase C activation and advanced glycation end product promoting inflammation and oxidative stress, as well as the mitochondrial dysfunction leading to bioenergetic failure was extensively reviewed in a recent publication [8]. It was acknowledged that DN is still lacking disease-modifying therapies and that the newer *omics* techniques development could play a critical role in understanding the mechanisms of nerve damage in diabetes, thus providing molecular targets for efficient treatments.

Metabolomics, a branch of omics sciences, focuses on the comprehensive profiling of metabolites in biological systems. Utilizing advanced analytical techniques like nuclear magnetic resonance (NMR) spectroscopy and mass spectrometry (MS), metabolomics provides a snapshot of the physiological state of an organism [9]. In research related to DN, metabolomics can reveal metabolic alterations, identify potential biomarkers for early diagnosis, and elucidate disease mechanisms. It offers insights into metabolic pathways affected by diabetes, such as altered glucose and lipid metabolism, which can contribute to neuropathic changes. This molecular insight supports the development of targeted therapeutic strategies and enhances the understanding of individual variability in disease progression and response to treatment, paving the way for precision medicine in diabetes management.

While genomic, transcriptomic, and proteomic studies have already been quite extensively reported in animal and human research in DN [10], the metabolomic studies in DN are less well-represented. Therefore, we sought to perform an extensive review and to critically examine the progress in metabolomic assessment in animal models and humans with DN.

## 2. Key Findings from Metabolomic Studies in DN

### 2.1. Energy Metabolism and Carbohydrates

Mitochondria plays an important role in energy homeostasis and its exposure to high glucose produces ultrastructural alterations that can be measured using metabolomic techniques, as was previously described in animal models and humans with diabetic neuropathy (DN) and neural crest culture cells exposed to high glucose. Chowdhury SK et al. observed that in rats with streptozotocin (STZ)-induced diabetes the changes in rates of coupled respiration with pyruvate+malate and with ascorbate+tetramethyl-p-phenylenediaminedihydrochloride (TMPD—tetramethyl-p-phenylenediaminedihydrochloride) did not occur in the first 12 weeks, but by 22 weeks of hyperglycemia exposure, respiration with pyruvate+malate was decreased and also with ascorbate+TMPD in the mitochondria from dorsal root ganglia. An important founding was that by administration of insulin these changes were significantly improved, independently of other indices of diabetes, such as glycated hemoglobin, that did not correct. Since the pathogenesis of this mitochondrial disorder might involve insulin deficiency per se, the authors suggested that insulin had direct effects on the mitochondrial function. The neurotrophic propensities of insulin beyond its fundamental role in glucose metabolism have been previously described [11]. Other significant modifications observed in rats with diabetes versus controls were decreased mitochondrial complexes I and IV and citrate synthase (the Krebs cycle enzyme), but with no increased reactive oxygen species in perikaryon (the cell body of the neuron, containing the nucleus and organelles). In addition, nerve fructose was increased in animals with diabetes and insulin therapy had no effect on this alteration [12]. Fructose accumulation results from too much glucose metabolized by aldose reductase in the polyol pathway, one of the crucial pathways described in DN development and progression [13].

Mitochondrial oxidative phosphorylation showed a striking upregulation in the distal sciatic nerve, but not in the corresponding cells of the cranial trigeminal ganglia or the lumbar 4/5 dorsal root ganglia, in a system-wide analysis of the peripheral nervous system of a rodent model of DN (*n* = 16). Multiple subunits of complexes I, IV, III, and V had increased expression; conversely, complex II displayed no changes. The metabolic dysfunction manifested as failure in energy homeostasis and/or oxidative stress was identified in the distal axon of the sciatic nerve leading the authors to conclude that distal followed by proximal distribution specific for DN was explained by the severity of molecular consequences [14].

Neural crest derived PC12 cells cultured in high glucose were used as a model for the development of DN and were analysed using proton nuclear magnetic resonance spectroscopy (1H-NMR) measurement. Their intracellular metabolites presented significantly different levels of 26 metabolites involved in energy metabolism, osmoregulation, membrane and methyl group metabolism, and amino acid metabolism compared to control cells. The metabolomic analysis indicated that the metabolic disturbances occurred both in intra- and extracellular media. Lactate levels, a metabolite with a key role in energy provision for the brain in addition to glucose during the hypoglycemic state, were decreased intracellularly and extracellularly. Succinate, a TCA cycle intermediate, and creatine phosphate, utilized for the formation of adenosine triphosphate (ATP) in conditions of high-energy demands, were both increased in PC12 cells exposed to high glucose. Authors suggested that energy metabolism was derived from glucose metabolism rather than lactate metabolism. In addition, a downregulation of the methyl group metabolism indicated by decreased intracellular 3-methylhistidine, dimethylamine, and dimethylglycine levels was found in high glucose conditions [15].

Xu W et al. studied 20 serum metabolites in T2DM patients with and without DN (*n* = 36) using 1H-NMR spectroscopy. Formate was identified as the only biomarker that was significantly decreased in the DN group after multiple comparisons and statistical adjustment. Endogenous production of formate occurs in mitochondria, where serine is catabolized to glycine and formate. It was also described as a mediator of metabolic interactions between host metabolism–diet–microbiome metabolism. Low formate concentrations in DN patients may be an indicator of mitochondrial dysfunction and gut microbiota alterations [16]. Formate can also be derived from dimethylglycine and sarcosine, intermediates in the choline catabolism. The authors suggested that decreased formate levels in the high glucose treated PC12 cells could been related to a reduction in dimethylglycine and speculated that a decrease in methyl group metabolism might also be responsible for PC12 cell death exposed to high glucose. Additional results using liquid chromatography–mass spectrometry (LC–MS), gas chromatography—mass spectrometry (GC–MS), genomic or proteomic analysis would have provided a more detailed metabolic picture of DN development [15].

In different animal models, metabolomic studies showed that the intermediates of glycolysis and the TCA cycle have different responses. In a mouse model (BKS.Cg-m+/+Leprdb; db/db) of T2DM, Hinder LM et al. evaluated the changes in glycolytic and TCA cycle metabolomes using targeted metabolomic analysis on dorsal root ganglia, sciatic nerve, and sural nerve. Decreased glycolytic and TCA cycle intermediates were found in the T2DM model after 20 weeks, with concurrent increases in protein and lipid oxidation. There was a decrease in four of the five measured glycolytic intermediates in the T2DM sural and sciatic nerves: glucose-6-phosphate/fructose-6-phosphate;hosphoenolpyruvate; 3-phosphoglycerate/2-phosphoglycerate and lactate; with additional decrease in fructose-1,6-bisphosphate in the sural nerve. Conversely, no changes were found in the dorsal root ganglia. Citrate and isocitrate, two TCA cycle metabolites, were significantly lower in all three tissues from the T2DM model, with a greater decrease seen in sciatic and sural nerves. Although the study showed data from the beginning of the characterisation of the bioenergetic alterations in DN, the metabolic impact of T2DM suggested by changes in the TCA cycle metabolome was greater distally, concomitant with proximal to distal increase in glycolytic metabolome [17]. In a T1DM animal model (produced by injecting STZ or alloxan which produce beta-cell deaths), the intermediates of glycolysis and the TCA cycle tended to increase, while in T2DM models (produced by various genetic defects causing insulin-resistance, hyperglycemia, dyslipidaemia, obesity and/or high fat diets) those metabolites tended to decrease [10]. Rojas et al. investigated the sequence of events occurring in metabolic pathways influenced in relation to sensory loss and nerve damage in the STZ model of T1DM analysed longitudinally up to 22 weeks. TCA, the main pathway of carbohydrate metabolism responsible for energy generation, was found impaired in T1DM and it was considered to be the primary cause of shunting metabolic substrate to compensatory pathways, leading to onset and progression of peripheral DN. In addition, ketoglutaric acid, citric acid, fumaric acid, succinic acid, and malic acid were found to be reduced in the sciatic nerve of T1DM. Conversely, sorbitol and L-lactate levels were increased in the peripheral nerve of the T1DM mice model [18]. Altered levels of 15 serum metabolites were significantly perturbed in patients with diabetes complications (*n* = 35), including DN, compared to diabetes without chronic complication (*n* = 38). The panel of metabolites included TCA cycle (succinate, citrate), methylamine metabolism (trimethylamine, betaine, methylamine), and energy metabolites (glucose, pyruvate, lactate) [19].

Zhao B et al. developed a targeted-metabolomic approach by measuring 56 metabolites using high-performance ion chromatography–tandem mass spectrometry (HPIC–MS/MS) to study carbohydrate metabolism in patients and animal models with DN. In patients with DN (*n* = 32) and controls (*n* = 25), 41 metabolites were successfully quantified, and 25 targeted metabolites showed significant changes in DN versus controls (18 increased and 7 decreased). The first five most significant changes were observed in the following: glucose 6-phosphate; cyclic 3′,5′-AMP, mannose 6-phosphate; isocitric acid; and glyceric acid. In animal models (rats with STZ induced diabetes), 52 targeted metabolites were measured in serum and 50 metabolites were measured in sciatic nerve tissue. The changes in DN rats (*n* = 18) versus controls were observed in 19 serum metabolites and 13 sciatic nerve metabolites. In both serum and sciatic nerves of the DN rats, the levels of 2-ketobutyric acid, hippuric acid, and 4-hydroxyphenylpyruvic acid were increased, while levels of picolinic acid, homogentisic acid, and quinolinic acid were reduced [20].

Xu J et al. conducted an ultra-high performance liquid chromatography coupled to tandem mass spectrometry (UHPLC–MS/MS)-based metabolomic study in patients with T2DM and DN (*n* = 60), T2DM without DN (*n* = 30), and healthy controls (*n* = 30). Using untargeted metabolomics, the authors identified 938 metabolites, and among them, the number of 12 metabolites was significantly higher in the DN group compared to the other groups. Correlation analyses showed that those metabolites were related to the changes in glucose metabolism and lipid metabolism. The 12 metabolites identified in this study were as follows: dimethylarginine; N-acetylhistidine; cysteine; 7-methylguanine; N6-acetyllysine; N,N,N-trimethyl-alanylproline betaine; N6-carbamoylthreonyladenosine; 5- methylthioadenosine; pseudouridine; aconitate; C-glycosyl tryptophan; and N2,N2-dimethylguanosine. In the authors opinion, these 12 metabolites could have pathogenetic implication in DNP [21].

Metabolomic analysis revealed significantly increased levels of 53 serum metabolites and 56 urine metabolites in T2DM and DN patients (*n* = 74) compared to T2DM patients (*n* = 41). The development of DN was associated with significant changes in the L-arabinose/L-arabitol ratio and four serum metabolic pathways of galactose metabolism, starch and sucrose metabolism, glyoxylate and dicarboxylate metabolism. The most significant urinary metabolites found to be increased in the T2DM and DN group were gluconic acid, erythritol, and galactonic acid. The development of DN was associated with the urinary metabolic pathways of the glyoxylate and dicarboxylate metabolism, the galactose metabolism, the ascorbate and aldarate metabolism [22].

### 2.2. Amino Acids, Peptides and Proteins

Zhang K et al. studied the metabolomic profile of (BKS Cg-m+/+Leprdb/J db/db) mice that developed DN at 16 weeks of age versus controls and observed that the biosynthesis of the amino acids (valine, leucine, and isoleucine), and the taurine and hypotaurine metabolisms were altered in DN mice [23]. The analysis of lumbar dorsal root ganglia and the sciatic nerve of mice, used as the T2DM model due to a leptin receptor mutation, indicated that several proteins grouped into functional clusters were differentially expressed compared to mice from the control group. Muscle-like proteins in dorsal root ganglia were upregulated, while those in the sciatic nerve were downregulated. Increased cytoskeleton-related proteins, a mild dysregulation of folding chaperones, activation of oxidative stress related proteins, and alterations in glutathione metabolism were also described. These data indicated that molecular alterations in the dorsal root ganglia and sciatic nerve were present before morphometric changes related to DN were developed [24].

In both T1DM and T2DM, patients were often observed with a decreased level of L-serine and increased levels of branched-chain amino acids (BCAA): leucine, valine, and isoleucine [25]. Clinically relevant consequences of L-serine deficiency in the development of DN are disturbances in the biosynthesis of sphingolipids, particularly ceramides and phospholipids [26]. L-Serine links multiple metabolic pathways: carbohydrate metabolism, protein synthesis, and sphingolipid synthesis. L-Serine can be produced in the brain, but in peripheral tissues is obtained from the liver and kidney via glycine and one-carbon metabolism. Genetic or chronic diseases characterized by low serum levels of L-serine and accompanied by neurological pathology and dietary interventions in animal models alleviate symptoms of DN. A serine tolerance test (STT) was developed in an animal model to assess L-serine homeostasis. Oral co-administration of 400 mg/kg L-serine and 2 g/kg glucose to mice drives an altered insulin response in STZ-treated mice (T1DM model) and BKS-db/db mice (T2DM model) versus healthy animals, with a decreased area under the curve (AUC) of L-serine and an increased AUC of glucose in mice with DM. Despite the higher amount of L-serine administered to heavier T2DM animals, the results supported the hypothesis that lower circulating L-serine levels in DM might have been the result of increased clearance and/or reduced absorption [27]. L-Serine was also found to be decreased in the brain of STZ-induced diabetic rats with the progression of DN at all time points, together with the following: L-threonine, L-methionine, D-proline, and N-acetyl-L-alanine. In addition, the amino acids precursor of analgesic neurotransmitters (L-tryptophan, L-histidine and L-tyrosine) were also downregulated in DN [28]. In patients with T2DM+obesity+DN, the levels of L-serine were lower, and L-alanine was higher versus controls (*n* = 75) [26]. Cardiovascular autonomic neuropathy in T1DM patients (*n* = 47) which was followed for 3 years correlated with lower baseline asparagine and glutamine and higher baseline fumarate levels (an intermediate in the citric acid cycle). Baseline higher serine, proline, threonine, phenylalanine, tyrosine, asparagine, aspartic acid, and histidine levels and lower fumarate levels were found in T1DM patients compared with controls. Also, baseline glutamine and ornithine levels were associated with the progression of cardiovascular autonomic DN after adjustment for the following confounding factors: diabetes duration, body mass index, baseline glycated hemoglobin, blood glucose, cholesterol, estimated glomerular filtration rate, urine microalbumin-to-creatinine ratio [29].

Building blocks of the BCAAs were observed to be associated with insulin resistance and development of diabetes and its related complications [30]. Most recently, Zhou ZY et al. observed that the levels of isoleucine, leucine, and valine in DN patients were significantly decreased by 15.09%, 13.37%, and 12.93%, respectively, compared to the levels of DM patients without DN. In the same study, the mouse model of DM confirmed that BCAA deficiency aggravated DN symptoms, whereas BCAA supplementation alleviated the symptoms. A potential explanation is that BCAA deficiency promotes neuropathic pain through upregulation of L-type amino acid transporter 1 and inhibition of Kv1.2 channel, which produces neuronal excitability [31]. In a T1DM mice model, BCAAs, valine, isoleucine, and leucine, increased more than twofold at very early post STZ administration. Similarly, serine, tyrosine, alanine, asparagine, histidine, and proline increased progressively with the diabetes progression [18]. Lin H.-T et al. used a 1H-nuclear magnetic resonance-based metabolomic technique to identify cerebrospinal fluid metabolomic profiles in T2DM patients versus controls. The results revealed a combination of alanine, leucine, valine, histidine, pyruvate, tyrosine and showed a significant correlation with the presence of DN (*n* = 11) [32].

In neural crest derived PC12 cells cultured in high glucose used as a model for the development of DN, the intracellular BCAAs, isoleucine and valine, were decreased, while glutamate was increased, suggesting that the hyperglycemia state increases the synthesis of glutamate from BCAAs. Higher alanine extracellularly could also have been responsible for increased glutamate. The authors suggest that there might be a connection between high glucose, high glutamate, and cell death. On the contrary, high levels of aspartate, another excitatory neurotransmitter, were decreased. Phenylalanine levels were increased intracellularly, while extracellular levels were reduced, corresponding with the theory that phenylalanine induced PC12 cell death in high glucose conditions [15].

In a clinical metabolomic study, 49 metabolites, previously associated with ocular and/or renal microvascular complications, were assessed in relation to the presence of DN. A number of 487 T1DM patients were enrolled, with biothesiometry available for 202 participants (77% had DN), followed-up for approximatively 5 years. The presence of microvascular complications was associated with eight metabolites (2,4-dihydroxybutanoic acid, ribonic acid, myoinositol, ribitol, 3,4-dihydroxybutanoic acid, valine, glycine, 2-hydroxyisovaleric acid) after adjustment for the following confounding factors: age, sex, duration of diabetes, body mass index, current smoking, systolic blood pressure, glycated hemoglobin, estimated glomerular filtration rate, total cholesterol, urinary albumin excretion rate, and statin treatment [33]. The presence of diabetes complications, including DN, indicated higher levels of valine, glutamate, methionine, arginine, proline, and threonine compared to patients with diabetes without DN [19].

β-alanine and the ratio of β-alanine/L-aspartic acid were the serum metabolites that displayed the most significant changes in T2DM patients with DN compared with their peers without DN. In addition, development of DN was associated with three serum metabolic pathways: aspartate, alanine, and glutamate metabolism; arginine biosynthesis; leucine, valine, and isoleucine biosynthesis. The most significant urinary metabolites found to be increased in the DN group were the following: cytidine, guanidoacetic acid, and aminoadipic acid. The occurrence of DN was associated with the following urinary metabolic pathways: arginine biosynthesis; arginine and proline metabolism; D-glutamine and D-glutamate metabolism; nitrogen metabolism; histidine metabolism [22].

The glucose time in range (TIR), defined as the percentage of time when the glucose values are between 70–180 mg/dL and measured using continuous glucose monitoring systems, was inversely associated with the devolvement of DN in T2DM patients (*n* = 105) [34]. Ma L et al. recently published an untargeted metabolomic study including 85 T1DM patients and 81 controls. Serum and urine samples were analysed by ultra-performance liquid chromatography–mass spectrometry and it was observed that T1DM patients had higher plasmatic levels of 5-methoxy-indole-acetate, 5-hydroxy-L-tryptophan, 4-pyridoxic acid, and 4-(2-aminophenyl)-2,4-dioxobutanoate. In urine samples, patients had higher urine concentrations of thromboxane B3 and lower urine levels of hypoxanthine versus controls. T1DM patients with TIR over 70% compared with T1DM with TIR below 50% had higher serum levels of mevalonolactone and 5-hydroxy-L-tryptophan and had higher urinary concentration of thromboxane B3 and phenyl-butyryl-glutamine. Further studies will explore if these metabolites have implications in the molecular pathways involved in the development of chronic complications of diabetes, including DN [35].

Asymmetric dimethylarginine (ADMA) is an endogenous inhibitor of nitric oxide synthase, having a potential role in the development of DN by increased oxidative stress damage, inflammation, fibrosis, and endothelial disfunction. Higher plasmatic ADMA levels are correlated with diabetes duration and are strongly associated with microvascular complications [36]. On the other hand, in a subgroup analysis of a meta-analysis consisting of 10 studies, patients with DN did not have a significant increase in circulating levels of ADMA [37]. Nevertheless, the role of ADMA in the development and progression of DN remains controversial.

Neurofilament light chain (NFL) is a polypeptidic cytoskeletal component involved in axonal stability of mature neurons. A multiplex approach using proximity extension assay technology was used to measure serum concentration in fasting conditions of NFL in 423 adults with T1DM and T2DM. It was observed that serum levels of NFL decreased with age and higher serum NFL was associated with DN (RR 1.92 per 1-normalised protein expression increase) after adjustment for age, sex, height, waist circumference, diabetes type and duration, glycated hemoglobin, cholesterol, estimated glomerular filtration rate, the presence of hypertension or cardiovascular disease, the use of non-steroidal anti-inflammatory drugs or lipid-lowering drugs [38].

Another method to identify the potential proteins involved in the development of DN is to identify the encoding genes that affect prone patients with diabetes to complications. In a study that enrolled 699 patients with T1DM/T2DM+DN and diabetic foot ulcers and 2695 controls (patients with T1DM/T2DM+DN, but without foot ulcers), the single-nucleotide polymorphism rs80028505 (Chr6p2131) in MAPK14 reached genome-wide significance. The protein encoded by this gene is involved in cellular response to proinflammatory cytokines [39]. A genome-wide association study investigating 6.8 million single nucleotide polymorphisms identified a cluster of 28 SNPs on chromosome 2q24 and found that the minor protective allele rs13417783 decreased the odds of DN odds by 36%. The presence of the protective allele was associated with higher expression in the tibial nerve of the SCN2A gene, which encodes a human voltage-gated sodium channel NaV1.2 [40]. The angiotensin-converting enzyme (ACE) is the enzyme that converts angiotensin I to angiotensin II, causing vasoconstriction, playing an important role in the pathogenesis of hypertension, diabetic nephropathy, and recently in DN. The ACE gene II genotype has a protective effect against DN in T2DM [41].

The proteomic and phospho-proteomic analyses of dorsal root ganglia (post-mortem sampling of tissue) of five donors with DN and five non-diabetic controls identified 6186 proteins, and 247 proteins were significantly different between groups. Out of these, after covariate control, 41 proteins and 27 phospho-peptides were identified after covariate control; 5 proteins and 20 phospho-peptides were regulated by fold change greater than 1.5, suggesting alterations of cellular structures and extracellular matrix remodelling [42]. Although Chowdhury SK et al. reported much earlier that in STZ diabetes-induced rats, nerve protein content was not influenced by diabetes, insulin therapy significantly increased this parameter in hyperglycemic animals and controls [12].

### 2.3. Lipids

In an animal model of db/db mice that developed DN at 16 weeks of age, it was observed that metabolisms of sphingolipid, unsaturated fatty acids, and arachidonic acid were modified. The sphingolipid metabolism was affected by a decreased level of five metabolites: sphinganine, sphinganine 1-phosphate, sphingosine 1-phosphate, phytosphingosine, and GlcCer (d18:1/16:0) [23].

Dysregulation of lipid metabolism, described as decreased palmitic (16:0), eicosanoic (20:0), and stearic (18:0) fatty acids found in the sciatic nerve, was less severe in the dorsal root ganglia and was not present in the trigeminal ganglia in an analysis of the peripheral nervous system in a rodent model of DN. Changes in structural and membrane lipids such as phospholipids, sphingolipids, and ceramides were also noticed in addition to alterations of the metabolic lipids. After the GC–MS analysis, nonpolar metabolomics was performed by LC–MS and revealed 397 unique metabolite features. Of these, 64.7% in the SN, 4.7% in the DRG, and 0.3% in the TG were significantly altered. The most altered lipids were the triacylglycerols, while the acylcarnitine species, linoleylcarnitine (18:2) and palmitoylcarnitine (16:0), were increased in the sciatic nerve [14]. In a human study, Song L et al. also found that metabolic disorders in DN included sphingolipid metabolism. In this study they analysed plasma and urine metabolites from patients with DM, DM+DN, and healthy controls and found 12, respectively 11 potential biomarkers for DN, among which were sphinganine and sphingosine [43]. Guo K et al. made an interesting observation in a study involving patients with obesity class III with peripheral neuropathy versus controls without neuropathy, matched by glycemic status (normoglycemia, prediabetes, or T2DM). They found that the diacylglycerol pathway discriminated obese individuals by neuropathy status, independent of glycemic status. Lipidomics also found other changes in phosphatidylcholine, sphingomyelin, ceramide, and dihydroceramide concentrations [44]. Among ceramides, 1-deoxysphingolipids was higher in T2DM+obesity+DN compared to controls (*n* = 75) and was inversely correlated with the intraepidermal nerve fiber density [26].

Higher lipid catabolism was revealed by the analysis of lumbar dorsal root ganglia and the sciatic nerve of BKS-Lepr db/db mice, used as the T2DM model due to a leptin receptor mutation. Several lipid catabolism-related proteins were upregulated (acetyl-CoA acetyltransferase, monoglyceride lipase, trifunctional enzyme, perilipin-1, enoyl-CoA delta isomerase I, Ras-related protein Rab-7a), while a number of proteins implicated in lipid biosynthesis were reduced (fatty acid synthase, 17-ß-HSD 12, NADP-dependent malic enzyme, and NADH-cytochrome b5 reductase 3) [24].

In a subcohort from the Danish arm of an Anglo–Danish–Dutch study of Intensive Treatment of Diabetes in Primary Care (ADDITION-Denmark) the presence of DN influenced 15 of the 991 total metabolites evaluated. In particular, two acylcarnitine species—lignoceroylcarnitine (24:0) and ximenoylcarnitine (26:1)—and one sphingolipid intermediate—glycosyl-N-(2-hydroxynervonoyl)-sphingosine (d18:1/24:1(2OH))—were significantly lower in DN. The results led the authors to the conclusion that the presence of DN changed the lipid abundance, chain length, and saturation for plasma free fatty acid and complex lipids, including sphingolipids, diacylglycerols, and acylcarnitines [45].

In a recent published study Zhong J et al. identified 1768 primary and secondary lipid metabolites in the serum of 60 patients with DM+DN and 20 controls. They observed significant differences between groups regarding the levels of sphingosine (d18:0), carnitine 22:1, lysophosphatidylethanolamine (LPE) (18:0/0:0), lysophosphatidylcholine (LPC) (0:0/18:0), LPC (16:0/0:0), (LPC) (18:1/0:0), and LPE (0:0/18:1). Furthermore, the levels of the first four metabolites were highly correlated with the patients’ electromyography results [46].

The serum metabolite caproic acid, a factor implicated in aggravating the inflammatory response in neurological diseases, and the urinary metabolic pathways of pantothenate and CoA biosynthesis were associated with the presence of DN in T2DM patients (*n* = 74) [22].

In a retrospective cohort study, Afshinnia F et al. examined lipidomic profiles of 69 T2DM patients, ten years prior to DN assessment, and observed a significant decrease of the serum medium-chain acylcarnitine level and increased total free fatty acids in DN cases. At baseline, patients with DN had also increased levels of lysophosphatidylcholines and decreased levels of phosphatidylcholines, independent of chain length and saturation, implying a prior potential change in lipidomics associated with DN development [47].

In an experimental study, metabolomic and lipidomic profiling of the spinal cord in T2DM rats with painful DN found that 45 lipids and a total of 170 other metabolites were dysregulated in the painful phase. Lipids, beyond their energy storage function, are indispensable components of cell membranes and cellular signal transduction. The top 20 lipidic metabolites that were different between the DN group and the non-DN group comprised the following: saccharolipid, phosphatidylcholines, phosphatidylethanolamine, triacylglycerols, ceramides, sulfated hexosylceramide and diacylglycerol [48]. In a human study, phosphatidylcholine C36:1, lyso phosphatidylcholine C18:1, and lyso phosphatidylcholine C18:2 were increased in T2DM+ DN (*n* = 27) versus T2DM patients without DN (*n* = 58) [49].

High-density lipoproteins (HDL) are emerging as determinants in the development of microvascular complications in T2DM, including DNP. In a cross-sectional, case-control study involving patients with T2DM and healthy subjects of Asian and Caucasian origin, the investigators analysed 32 HDL fractions and subfractions. After different adjustment models including age, sex, and current smoking status, the lower levels of lipid content in the smallest HDL (HDL-4) in patients of Asian origin with T2DM seems to have higher odds of being linked with microvascular complications, such as DN [50].

Another structure involved in lipid metabolism and studied as a potential factor implicated in DN is apolipoprotein-CIII (apo-CIII). It is associated with an increased risk of microvascular complications in diabetes via inhibition of triglyceride clearance from plasma. There are four proteoforms: a non-glycosylated apo-CIII and three O-glycosylated forms zero-, mono-, or di-sialyated apo-CIII. Naber A et al. observed that higher levels of mono-sialylated apo-CIII measured by mass spectrometry of the intact protein in the plasma were associated with neuropathy in a cohort of T2DM patients [51].

### 2.4. Therapeutic Approaches

The use of metabolomics may discover new pathways involved in DN and identify new therapeutic targets. The polyol pathway is one of the most studied pathogenic mechanisms for development of DN [14], but in aldose-reductase knockout mice and wild-type mice with STZ-induced diabetes, an elevated glucosamine level was observed. Furthermore, infusion of glucosamine for 12 weeks using a subcutaneous pump induced nerve fiber loss in the epiderma and slowing of nerve conduction, suggesting glucosamine as a potential toxic factor and candidate for a therapeutic target. Other upregulated substances found in this study were galacturonic acid, sarcosine, isobutylamine, and NADP+ [52]. The neuroprotective effect of nicotinamide riboside, a precursor of NADP+ and NADPH added to the high fat diet of the mice model of T2DM, was measured by quantitative metabolomics. Although the hepatic levels of NADP (+) and NADPH were significantly degraded in T2DM with sensory neuropathy (*n* = 20), they were significantly protected by the supplementation of nicotinamide riboside [53].

Taurine supplementation has no effect on weight or glucose values in STZ-induced diabetic rats. However, taurine increased serum malondialdehyde and decreased neuroinflammation by activating Nrf2 and suppressing NF-κB expression, with beneficial effects on diabetic neuropathy [54]. Melatonin supplementation at doses of 3 and 10 mg/kg might also have positive results in experimental diabetic neuropathy, due to the effects on NF-kB that reduce neuroinflammation, and Nrf2 cascades that reduce oxidative stress [55]. Another new option for the treatment of DN might be serine supplementation, but until now there is evidence only for prevention of the onset and progression of experimental DN. Further studies will be necessary to evaluate the clinical significance of serine supplementation in alleviating the symptoms of DN or reducing the progression of already developed DN in DM patients [56].

Carnosine (β-alanyl-l-histidine) is an imidazole dipeptide found in the nervous system, muscles, and other tissues with antioxidant, antiglycation, metal chelator, and hydroxyl radical scavenger function. At the metabolomic level, it has been found to have neuroprotective effects against neurodegenerative diseases by increasing the secretion of neurotrophins, such as brain-derived neurotrophic factor and nerve growth factor [57]. The supplementation with carnosine as add-on therapy to vitamin B complex in T2DM with neuropathy alleviated symptoms of DN after 12 weeks of therapy [58].

JinMaiTong (JMT) is a traditional Chinese medicine compound used for treating DN. The use of JMT decoction for 12 weeks in rats with STZ-induced diabetes extensively regulated the metabolic disturbance in DN and improved the pain threshold, increased the nerve fiber density, and positively influenced the axonal demyelination and atrophy of the sciatic nerve. A number of 21 metabolites were identified: 16 biomarkers were found in both the JMT-high dose and JMT-low dose treated groups, while the five others were specific to the JMT-high dose group. The previously mentioned metabolites are involved in amino acid metabolism, lipid metabolism, and the tricarboxylic acid cycle. Metabolites in the lipid metabolism were the following: glycerophospholipid metabolites, carnitine and triacylglycerols. Also, JMT decoction affected seven metabolic pathways: glyoxylate and dicarboxylate metabolism; aspartate, alanine, and glutamate metabolism; glycerophospholipid metabolism; tricarboxylic acid cycle; glycerolipid metabolism; and pyruvate metabolism [59]. Several years later, the administration of JMT to DN rats for 6 weeks improved the neurological function even after demyelination and axonal degeneration. JMT significantly increased the serum concentrations of citric acid (the Krebs cycle), gluconic acid and glyceric acid (the pentose–phosphate pathway), and adenosine and guanosine triphosphate (important energy donors for cellular function). On the other hand, JMT reduced α-ketoglutaric acid and succinic acid (the Krebs cycle). In sciatic nerve tissues, administration of JMT increased the level of glucose 1-phosphate and decreased the levels of 3-phosphoglyceric acid and L-lactic acid (regulating the saturated glycolytic pathway caused by hyperglycemia). It also decreased two precursors of Krebs cycle metabolites (2-hydroxyglutaric acid and 2-ketobutyric acid), implying a possible role in regulation of mitochondrial function. In cultured rat Schwann cells exposed to hyperglycemia, JMT activated adenosine monophosphate-activated protein kinase (AMPK) and downstream peroxisome proliferator-activated receptor gamma coactivator 1-alpha (PGC-1α), restoring the energetic profile of the cell [20].

Another traditional Chinese herbal formula with potential therapeutic effect in DN is Huangqi Guizhi Wuwu Decoction (HGWD). BKS Cg-m+/+Leprdb/J mice (db/db mice) developed DN at 16 weeks of age and received 8 weeks of treatment. Administration of HGWD in DN mice improved peripheral nerve structure and serum lipid profile, enhanced antioxidant capacity, reduced inflammation, increased the serum level of nerve growth factor and reversed shift plasma metabolic profiles. In this study, 37 metabolites were significantly different among controls, DN mice, and the HGWD treatment group. The use of HGWD ameliorated four pathways: sphingolipid metabolism, synthesis of unsaturated fatty acids, metabolism of arachidonic acid, and synthesis of three amino acids (valine, leucine, and isoleucine) [23]. Using HPLC–MS/MS, 11 chemical compounds of HGWD were identified. The reduction of oxidative stress by HGWD (Tang Luo Ning recipe) was comparable with the administration of alpha-lipoic acid and was driven through Nrf2 (nuclear factor erythroid-derived 2-like 2) and Bcl2 (family of proteins that regulate cell survival) activation in the sciatic nerve [60].

The potential therapeutic role of omega-3 fatty acids (1000 mg docosahexaenoic acid/200 mg eicosapentaenoic acid) was investigated in a study that enrolled 40 patients with T2DM. The study duration was 3 months and included metabolomic analysis of plasma samples. The conclusion was that omega-3 fatty acid supplementation had the potential to reduce neuropathic pain in T2DM patients and correlated with plasmatic sphingosine levels [61]. Several years later, the team conducted by Duran described the distinct cellular pathways elicited by dietary omega-3 PUFA supplementation in patients with T2DM affected by painful DN. The supplementation induced an increase in several metabolites involved in oxidative stress and glutathione production: glycine cysteine-glutathione disulfide (+157%), cystathionine (+90%), cysteinylglycine (+19%), arginine (+13.4%), glycine (+11%), S-methylmethionine (+9%), while glutamate (−11%) decreased. Also, linoleoyl-docosahexaenoyl-glycerol (+253%), a phospholipid associated with improved membrane fluidity, was significantly increased. Thus, the study investigators concluded that dietary omega-3 PUFA supplementation reduced the pro-inflammatory and oxidative stress pathways associated with neuroinflammatory diseases, emphasizing the promising role of these fatty acids in reducing the symptom intensity in painful neuropathy [62]. In mice model, dietary enrichment with omega-3 polyunsaturated fatty acids attenuated nociceptive behaviours and neurophysiologic abnormalities induced by a high omega-6 polyunsaturated fatty acid enriched Western-style diet during an 8-week time period. The persistent nociceptive hypersensitivity was related to the accumulation of linoleic and arachidonic acids in lumbar dorsal root ganglia and skin [63].

Bupropion is an antidepressant drug that has the potential to counteract glucotoxicity in Schwann cells. In hyperglycemic conditions, glycerolipid metabolism enzymes in Schwann cells are upregulated and bupropion treatment produced changes in the enzymes and metabolites of this metabolic pathway. In animal models and in vitro studies bupropion administration prevented sensory disfunction, Schwann cell death, and damage of myelin [64].

Serger E et al. reported that indole-3-propionic acid, produced in human gut by the gram-positive bacteria Clostridium sporogenes, especially in intermittent fasting condition, promoted efficient axonal regeneration after sciatic nerve crush in mice, involving an immune mediated mechanism. It remains to be established if this mechanism has therapeutic potential in the treatment of DN [65].

## 3. Conclusions and Further Perspectives

Using different and complex techniques, metabolomics has emerged as an important approach for investigating, identifying, and describing biomarkers related to DN. A number of compounds were found to be associated with DN but a remarkable variability among reported results should be noted. The reproducibility of this type of studies is burdened by variability factors such as the collection and pre-processing of biological samples, the lack of a harmonization between laboratories, as well as the existence of an inter-individual variability of the metabolome that was more recently recognized [66]. These challenges in metabolomic studies could explain the fact that none of the identified biomarkers has yet been validated for use in clinical practice. The ideal metabolite or set of metabolites that could be used as biomarkers should identify patients with diabetes prone to develop DN or those prone to progress to severe forms of sensory loss associated with risk of ulcerations and amputation. Another potential use of a metabolite might be as an indicator of treatment response in clinical trials using agents with potential disease-modifying properties.

## Data Availability

No new data were created or analyzed in this study.
